# Sex-specific associations between sleep apnoea and lung cancer risk in patients with COPD: a nationwide prospective cohort study

**DOI:** 10.1016/j.lanepe.2025.101269

**Published:** 2025-03-20

**Authors:** Kristiaan Proesmans, Annemarie I. Luik, Lies Lahousse

**Affiliations:** a Department of Bioanalysis, Faculty of Pharmaceutical Sciences, Ghent University, Ottergemsesteenweg 460, Ghent, 9000, Belgium; b Department of Epidemiology, Erasmus Medical Center, Doctor Molewaterplein 40, Rotterdam, 3015, the Netherlands; c Trimbos Institute, The Netherlands Institute of Mental Health and Addiction, Utrecht, the Netherlands

**Keywords:** Sleep apnoea, COPD, Lung cancer

## Abstract

**Background:**

COPD is an established risk factor for lung cancer. Sleep apnoea is prevalent in COPD and the inflammation caused by intermittent hypoxaemia may increase this lung cancer risk. Females have more systemic inflammation for a similar apnoea-hypopnoea index than males. Therefore, this study aims to investigate sex-specific associations between sleep apnoea and lung cancer in COPD.

**Methods:**

The sex-specific absolute and relative risk of sleep apnoea on newly diagnosed lung cancer was estimated in a nationwide observational study of Belgian patients with COPD (≥55 years), between 2017 and 2022, using an Aalan-Johanson estimator and a cause-specific Cox regression model adjusted for age, socioeconomic status, smoking status, alcoholism, frailty, comorbidities, and comedication.

**Findings:**

The study consisted of 62,903 COPD patients (42·80% female), of whom 2898 (4·60%) developed lung cancer. We found a significant sex interaction of sleep apnoea on lung cancer hazard (***χ***-squared: 13·239, P-interaction < 0·01). In females, sleep apnoea was associated with a higher lung cancer risk (cumulative incidence: 1545 vs 1350 per 100,000 PY; aHR: 1·31 (95% CI: 1·05–1·63)). For males, sleep apnoea patients had a lower lung cancer risk (cumulative incidence: 1632 and 2305 per 100,000 PY; aHR: 0·82 (95% CI: 0·70–0·95)). The impact of sleep apnoea on lung cancer development was especially strong in female COPD patients with hypoxia-related comorbidities e.g., with a history of emphysema (aHR: 2·65 (95% CI: 1·11–6·34)).

**Interpretation:**

Sleep apnoea was associated with a higher risk of lung cancer in female COPD patients while, in males, there was a lower risk. Especially in female COPD patients with hypoxia, sleep apnoea is strongly associated with an increased lung cancer risk.

**Funding:**

Emmanuel van der Schueren cancer research fellowship “10.13039/501100011851Kom Op Tegen Kanker”.

**Research in context**

**Evidence before this study**

We searched PubMed using the Mesh terms “Pulmonary Disease, Chronic Obstructive”, “Sleep Apnoea Syndromes”, and “Lung Neoplasms” for articles published between Jan 1, 2010 and November 30, 2024. We found no studies that measured the impact of sleep apnoea on the risk of lung cancer, in COPD patients. Remarkably, recent research found a high prevalence of sleep disturbance breathing in lung cancer and in COPD patients.

Afterwards, we broadened the search, using only the Mesh terms “Sleep Apnoea Syndromes” and “Lung Neoplasms”. A 2024 review concerning obstructive sleep apnoea and lung cancer reported that seven of thirteen studies, including two meta-analyses, found a positive association. However, four studies did not find an association and two studies even found a negative association. The negative association was especially evident in males. Major heterogeneities in study design and populations make it impossible to conclude an overall increased lung cancer risk by sleep apnoea.

**Added value of this study**

To the best of our knowledge, this is the first study to investigate the link between sleep apnoea and lung cancer in a COPD population. We observed an increased risk of lung cancer in females, while a protective effect was found in males. An increased hazard was especially evident in females with hypoxia-related comorbidities. Our results give insight into the complex relationship between sex hormones, intermittent hypoxia, and lung cancer. Clarifying the heterogenic link between sleep apnoea and lung cancer.

**Implications of all the available evidence**

Intermittent hypoxia and sex hormones play a major role in the association between sleep apnoea and lung cancer. Future research could further explain these underlying mechanisms, explaining the differential underpinnings towards lung cancer. Providing a foundation for more effective personalised lung cancer prevention strategies.

## Introduction

Lung cancer is currently the second most diagnosed cancer and the leading cause of cancer-related deaths worldwide.^1^ While smoking remains the principal risk factor, compelling evidence shows previous respiratory diseases to be an independent risk factor as well.^2^

Chronic obstructive pulmonary disease (COPD) and sleep apnoea are two common obstructive lung diseases of respectively lower and upper airways. COPD is generally characterised by airflow limitation and persistent respiratory symptoms.^3^ Sleep apnoea is a sleep-disorder causing repeated lapses in breathing. These are mainly caused by an upper airway occlusion (obstructive sleep apnoea), or more seldom by a transient reduction of the pontomedullary pacemaker in the generation of breathing rhythm (central sleep apnoea).^4^ Given the high prevalence of sleep apnoea, around 10–30% of people suffering from COPD may also have sleep apnoea.^5^ The co-occurrence of COPD and sleep apnoea is also referred to as the overlap syndrome.

Independent of smoking history, COPD confers a 2–7 times higher risk of lung cancer.^6^ Moreover, emphysema further increases this risk.^7^ In sleep apnoea, the link with lung cancer is less explored. Epidemiological studies linking sleep apnoea with lung cancer are suggesting an increased risk.^8^, ^9^, ^10^ Sleep apnoea increases the burden of systematic inflammation due to factors like disturbed sleep, intermittent hypoxia, and oxidative stress.^8^^,^^11^ These inflammatory processes enhance lung tumorigenesis, making it likely for sleep apnoea to increase lung cancer risk.^8^ Especially hypoxia has been described to fuel tumorigenesis.^11^ On the contrary, recent findings have found a protective effect of sleep apnoea on lung cancer, particularly in males.^12^^,^^13^ Sex differences have been described, as females with sleep apnoea have more systematic inflammation for a given apnoea-hypopnoea index and are more likely to suffer from pulmonary hypertension.^14^^,^^15^ Furthermore, sleep-disordered breathing is often underdiagnosed and undertreated in females compared to males.^14^ This indicates that the impact of sleep apnoea on lung cancer might have sex-specific underlying mechanisms that require sex-specific management.

In this nationwide prospective research based on insurance records and hospital registries, we aimed to investigate the role of sleep apnoea in the development of lung cancer and we hypothesised that lung cancer risk would be especially increased in female COPD since they suffer more hypoxia than males with similar sleep apnoea severity.

## Methods

### Data source

Data from the Intermutualistic Agency (IMA) was combined with the Minimal Hospital Dataset (MHD). The IMA dataset contains all claims from Belgian insurance funds on reimbursed ambulatory and hospital care. Health insurance is obligatory in Belgium. The dataset comprises demographic characteristics, drug (outpatient) prescription claims, and medical procedures including diagnostic, therapeutic procedures, and other reimbursed care. The MHD contains all hospital discharge diagnoses and inpatient medication of each hospital admission in Belgium. This includes hospitalisations, day-care stays, and emergency room contacts. All diagnoses are reported by the International Classification of Diseases (ICD) codes (ICD-9 up to 2014, ICD-10 from 2015 onward) and researchers only had access to a pseudonymized linked dataset. This study was approved by the IMA and MHD database administrators and by the “Social Security and Health Chamber of the Belgian Information Security Committee” (approval code IVC/KSZG/23/008). This study aligned with the Strengthening Reporting of Observational Studies in Epidemiology (STROBE) reporting guideline (eTable S1).

### Study population

The COPD population was defined as patients having disease codes J40-J44 codes (ICD-10) and chronically using obstructive airway medication (i.e., at least 2 filled prescriptions of R03 classified medications following the anatomical therapeutic chemical classification system (ATC) within 1 year, including all currently available inhalers in addition to oral therapies specifically to treat asthma or COPD) aged ≥55 years, between January 1, 2017 and December 31, 2021. Patients with a cancer diagnosis before the index date (look-back period of 1 year) were excluded. A protocol scheme is shown in the Supplementary File (eFigure S1).

### Exposure and outcome

The exposure of interest was sleep apnoea. This was defined by the G47·3 code (ICD-10) or a medical procedure code related to sleep apnoea (e.g., CPAP usage) at baseline, described in the Supplementary (eTable S2). CPAP users are required to have a yearly check-up to evaluate therapy adherence, ensuring the validity of these codes. COPD patients were followed from the moment they met the COPD definition up until one of the following outcomes of interest occurred: lung cancer diagnosis, death, emigration, or end of study period (December 31, 2021), whichever came first.

### Covariates

Baseline characteristics were defined based on medical procedure codes, ICD-coded diagnoses, medical prescription codes, and/or medication prescription history within one year before the index date (eTable S2). Covariates were selected based on risk factors for lung cancer and sleep apnoea in the literature. The causal pathways are shown by a directed acyclic diagram in the Supplementary Files (eFigure S2). Analyses were adjusted for demographics including age, sex, socioeconomic status (which was approximated by the medical reimbursement of a person, in case the person received a higher medical reimbursement, the person is considered to have a lower socio-economic status, otherwise a regular socio-economic status is assumed), smoking, alcohol use disorder, a history of pneumonia, asthma, peripheral vascular disease, hypertension and diabetes, additional specific risk scores were calculated for frailty, and the Charlson comorbidity index (without accounting COPD), and medication usage of inhaled corticosteroids, sedatives, and hypnotic drugs was taken into account. Smoking (active smoker, past smoker, or no smoker) was defined by smoking-related ICD-codes and therapy related to smoking cessation, registered since January 1st, 2010 (eTable S2). Furthermore, stratifications were performed for hypoxic-related comorbidities (anaemia, cerebrovascular disease, chronic kidney disease, emphysema, and myocardial infarction, eTable S2) to explore potential aggravation of underlying mechanisms and for sleep apnoea treatment (oxygen therapy and CPAP usage, eTable S2) to explore potential relieve of underlying mechanisms.

### Statistical analysis

The characteristics of the study population were described by the median and interquartile range (IQR) for continuous variables. Categorical variables were shown as counts (n) with percentages (%). Differences between categorical variables were estimated by ***χ***-squared test. For continuous variables, differences were estimated by t-tests. The cumulative incidence function was estimated by an Aalen-Johansen estimator, accounting for the competing risk of death. Patients who emigrated or reached the end of the study period were censored. Furthermore, the cumulative incidence per 100,000 person-years (PY) follow-up was estimated for the total population and sex-specific subgroups, by dividing the number of new lung cancer cases by the follow-up period. The relative risks were estimated by univariable and multivariable cause-specific Cox models. In the multivariable model, interaction terms between smoking and sleep apnoea, and the usage of sedatives and hypnotics and sleep apnoea were tested. However, as they were not significant, the terms were not included. The proportional hazard assumption was visually checked. Specific subgroup analyses were performed by stratifying sex-specific subgroups by the occurrence of hypoxia related signs. A ***χ***-squared likelihood comparison estimated the significance of sleep-apnoea interactions with biological sex on lung cancer hazard.

### Sensitivity analysis

Given that incident sleep apnoea diagnosis during follow-up could lead to biased hazard estimations, relative risk analyses were repeated with the exclusion of patients with a (first) sleep apnoea diagnosis during follow-up. Additionally, the Cox model was repeated with a time-dependent variable for incident sleep apnoea and with the censoring of the first three months after the index date. Furthermore, specific subgroup analyses were performed to check the robustness of our conclusion. This included a subgroup analysis of people who also had a lung function test performed at baseline, people who received triple therapy to treat COPD, people who received a specific J44 ICD-10 code, people who did not smoke, and people who had ever smoked, a subgroup with only and a subgroup without people who use a CPAP among the sleep apnoea cases.

### Ethics statement

The study received approval from the InterMutualistic Agency database and Minimal Hospital Dataset administrators, as well as the “Social Security and Health Chamber of the Belgian Information Security Committee (approval code IVC/KSZG/23/008). In accordance with national legislation and institutional requirements, written informed consent for participation was not required.

### Role of the funding source

The funding source had no role in the study design, data analysis, interpretation, or writing of the study.

## Results

### Characteristics

A total cohort of 62,903 COPD patients were included in Table 1. The median follow-up time was 914 days (Q1–Q3: 389–1461, min-max: 1–1796). Among sleep apnoea patients, males were more likely to use a CPAP, compared to females (males: 2018/5210 (38·73%) vs females: 642/2434 (26·37%); ***χ***-squared: 111·10, P-value < 0·001). During follow-up, 2898 (4·60%) patients received an incident lung cancer diagnosis. Among males, the percentage of patients who received a lung cancer diagnosis in follow-up was lower among those with sleep apnoea compared to patients without sleep apnea (sleep apnoea: 219/5210 (4·20%) vs no sleep apnoea 1720/30,773 (5·59%); ***χ***-squared: 16·51, P-value < 0·001).

**Table 1:** Baseline characteristics, stratified by sleep apnoea and sex.

	No sleep apnoea		Sleep apnoea		Total, n (%)
	Male, n (%)	Female, n (%)	Male, n (%)	Female, n (%)	
Age category (years)					
55–64	5344 (17·4)	5051 (20·6)	1375 (26·4)	715 (29·4)	12,485 (19·8)
65–74	9765 (31·7)	7748 (31·6)	2182 (41·9)	919 (37·8)	20,614 (32·8)
75–84	9926 (32·3)	6722 (27·5)	1323 (25·4)	602 (24·7)	18,573 (29·5)
85+	5738 (18·6)	4965 (20·3)	330 (6·3)	198 (8·1)	11,231 (17·9)
Lower SES	12,728 (41·4)	12,317 (50·3)	1961 (37·6)	1308 (53·7)	28·314 (45·0)
Alcohol use disorder	5670 (18·4)	2651 (10·8)	977 (18·8)	241 (9·9)	9·539 (15·2)
Obesity or overweight	6326 (20·6)	5188 (21·2)	3138 (60·2)	1485 (61·0)	16·137 (25·7)
Smoking status					
No smoker	4529 (14·7)	6624 (27·1)	511 (9·8)	538 (22·1)	12·202 (19·4)
Past smoker	12,316 (40·0)	6036 (24·7)	2367 (45·4)	780 (32·0)	21·499 (34·2)
Current smoker	13,928 (45·3)	11,826 (48·3)	2332 (44·8)	1116 (45·9)	29·202 (46·4)
Asthma	2902 (9·4)	3541 (14·5)	666 (12·8)	493 (20·3)	7·602 (12·1)
Pneumonia	8276 (26·9)	5618 (22·9)	1255 (24·1)	556 (22·8)	15·705 (25·0)
Hypertension	20,285 (65·9)	16,272 (66·5)	4038 (77·5)	1828 (75·1)	42·423 (67·4)
Diabetes mellitus	10,272 (33·4)	7357 (30·0)	2746 (52·7)	1188 (48·8)	21·563 (34·3)
Peripheral artery disease	6860 (22·3)	3400 (13·9)	1085 (20·8)	302 (12·4)	11·647 (18·5)
CCI ≥ 4	21,282 (69.2)	15,269 (62.4)	3450 (66.2)	1502 (61.7)	41·503 (66·0)
Frailty	9625 (31·3)	9798 (40·0)	1105 (21·2)	788 (32·4)	21·316 (33·9)
Sedatives and hypnotic drugs	11,325 (36·8)	10,311 (42·1)	1972 (37·9)	990 (40·7)	24·598 (39·1)
ICS usage	23,055 (74·9)	19,561 (79·9)	3954 (75·9)	1972 (81·0)	48·542 (77·2)
CPAP usage	–	–	2018 (38·7)	642 (26·4)	2·660 (4·2)
Oxygen therapy	2530 (8·2)	2265 (9·3)	937 (18·0)	657 (27·0)	6·389 (10·2)
Emphysema	1254 (4·1)	830 (3·4)	170 (3·3)	66 (2·7)	2·320 (3·7)
Anaemia	5875 (19·1)	4794 (19·6)	880 (16·9)	455 (18·7)	12·004 (19·1)
Myocardial infarction	4712 (15·3)	2156 (8·8)	786 (15·1)	228 (9·4)	7·882 (12·5)
Cerebrovascular disease	5663 (18·4)	3968 (16·2)	877 (16·8)	355 (14·6)	10·863 (17·3)
Chronic kidney disease	6725 (21·9)	4778 (19·5)	1208 (23·2)	557 (22·9)	13·268 (21·1)

Categorical variables are presented as numbers (%). Continuous variables are presented as mean (SD). CCI: Charlson comorbidity index; ICS: inhaled corticosteroids; SES: Socioeconomic status.

### Absolute lung cancer risk

**Fig. 1:**
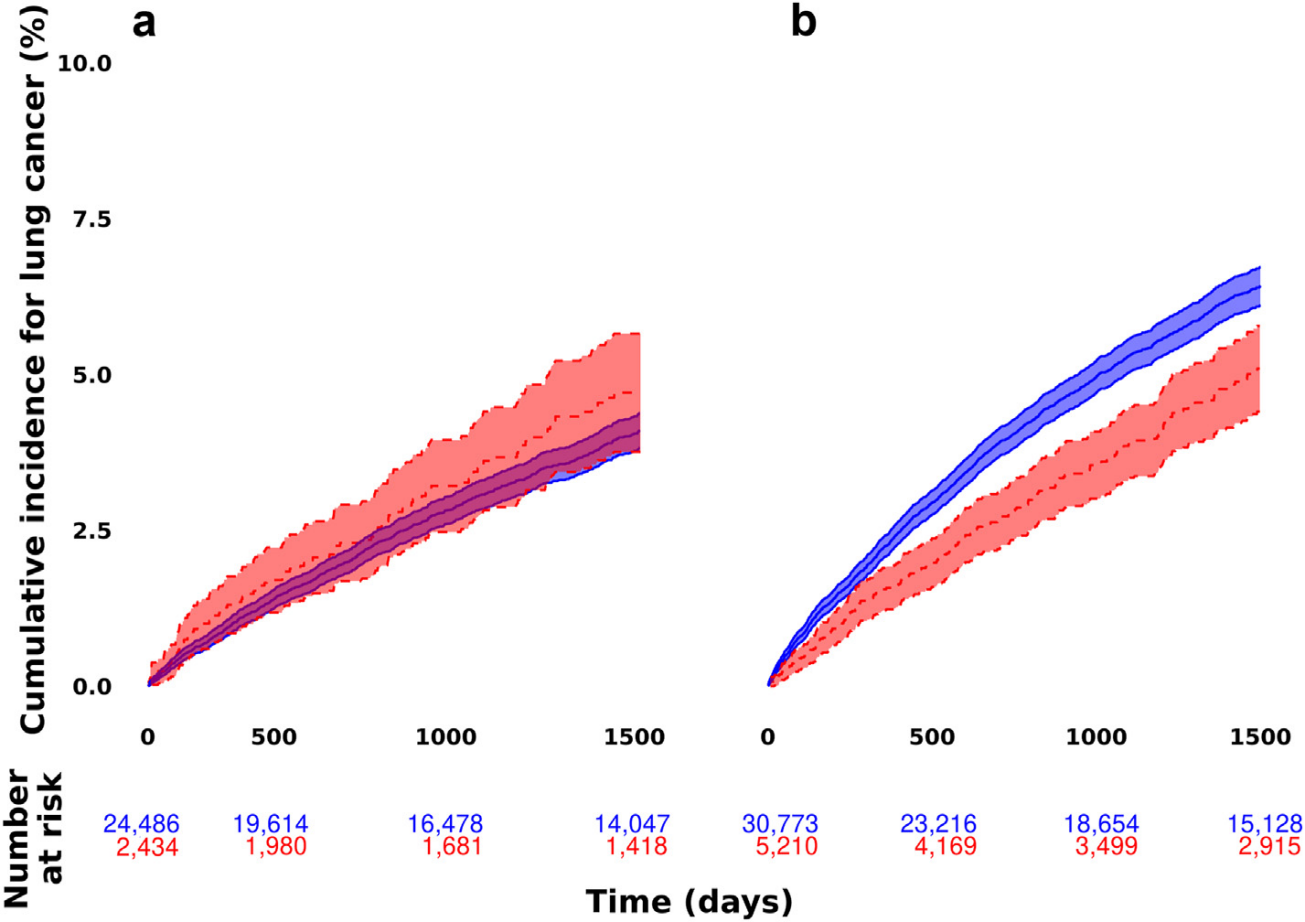
**Cumulative incidence of lung cancer in COPD patients.** The cumulative incidence of lung cancer in COPD patients with (dotted and red), or without sleep apnoea (solid and blue), stratified by sex. Panel a shows the cumulative incidence in females, panel b in males.

The sex-stratified cumulative incidence of lung cancer is illustrated in Fig. 1. Among female COPD patients, 860 (3·51%) patients without sleep apnoea received a lung cancer diagnosis during follow-up, while 99 (4·07%) patients with sleep apnoea received a lung cancer diagnosis. The cumulative incidence per 100,000 person-years, was 1545 (95% CI: 1257–1877) when there was a sleep apnoea diagnosis and 1350 (95% CI: 1262–1443) among female COPD patients without sleep apnoea.

In contrast, among male COPD patients without sleep apnoea, there were 1720 (5·59%) incident lung cancer cases. Among male COPD patients with sleep apnoea, there were 219 (4·20%) cases. The cumulative incidence per 100,000 person-years was 2305 (95% CI: 2198–2415) among male COPD patients without sleep apnoea and 1632 (95% CI: 1424–1861) for those with sleep apnoea.

In the total study population, the cumulative incidence of lung cancer per 100,000 person-years was respectively 1866 (95% CI: 1795–1938) and 1604 (95% CI: 1434–1789) for those without and with sleep apnoea.

### Adjusted relative lung cancer risk

**Fig. 2:**
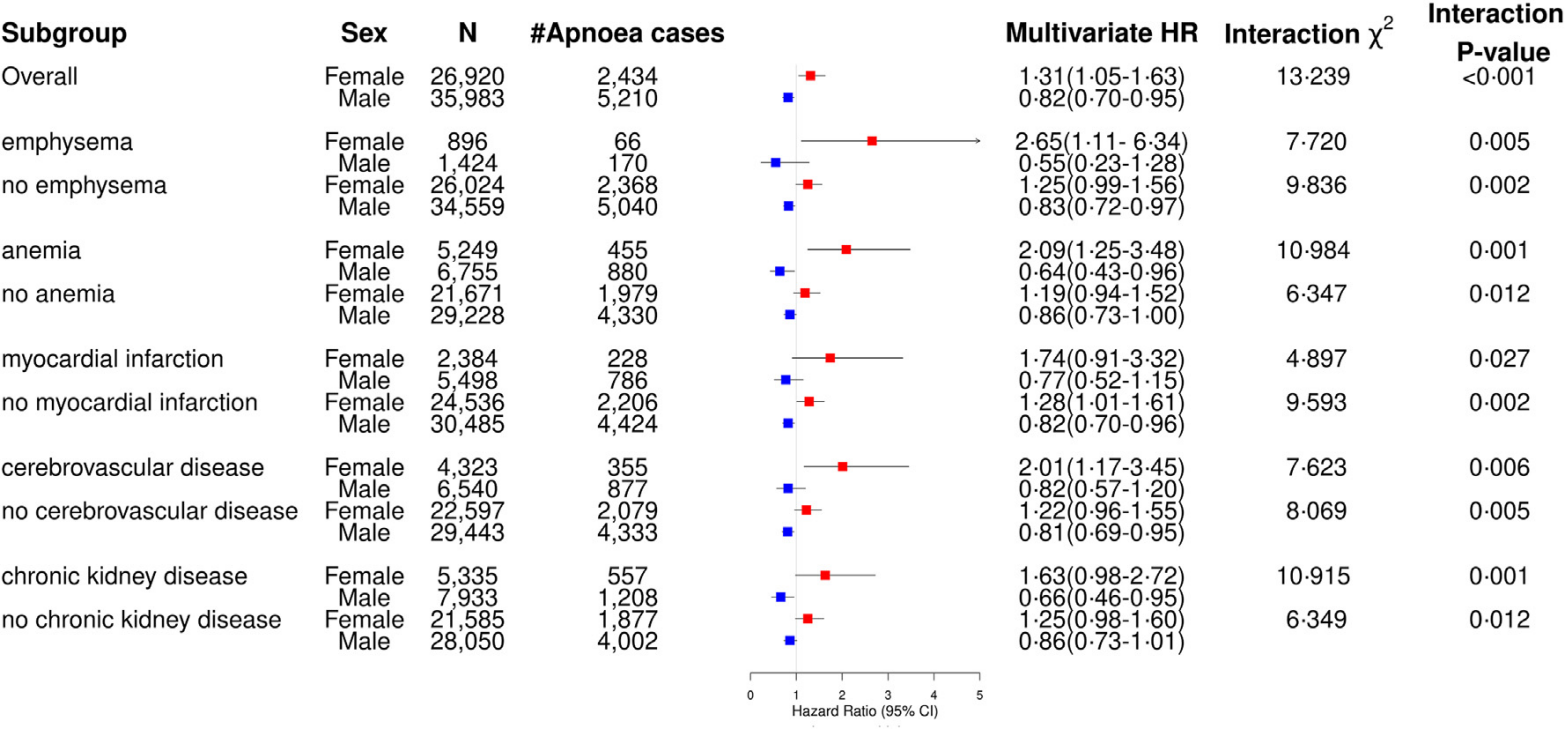
**The hazard ratio of sleep apnoea on lung cancer, in COPD patients.** The sex-specific hazard ratio of sleep apnoea on incident lung cancer, in COPD patients, adjusted for age, socioeconomic status, smoking status, alcoholism, overweight or obesity, hypertension, diabetes, asthma, pneumonia, peripheral vascular disease, Charlson comorbidity score, frailty, the use of inhaled corticosteroids, sedatives or hypnotic drugs. A sex-specific stratification is shown with females (red), and males (blue) for the association in the overall study population and in specific subgroups based on the presence of hypoxia-related comorbidities. The ***χ***-squared and P-values present the significance of the interaction between sexes and sleep apnoea on lung cancer hazard within each group.

Biological sex significantly modified the incident lung cancer hazard ratio related to sleep apnoea in univariable analysis (***χ***-squared: 13·196, P-interaction < 0·001, eFigure S3) and multivariable analyses accounting for age, socioeconomic status, smoking, alcohol use disorders, overweight or obesity, hypertension, diabetes, asthma, pneumonia, peripheral vascular disease, Charlson comorbidity index (CCI), frailty, the use of ICS, sedatives or hypnotic drugs (***χ***-squared: 13·239, P-interaction < 0·001, Fig. 2). Sleep apnoea was significantly associated with a higher hazard in developing lung cancer among females with COPD (aHR: 1·31 (95% CI: 1·05–1·63)), while a lower hazard of incident lung cancer in male COPD patients was observed (aHR: 0·82 (95% CI: 0·70–0·95)). Fig. 2 shows that sex significantly modified incident lung cancer hazard in all subgroup analyses by hypoxia-related comorbidities among COPD patients. Among the subgroup of female COPD patients with emphysema, anaemia, or cerebrovascular disease, sleep apnoea had a significantly increased lung cancer hazard (respectively aHR: 2·65 (95% CI: 1·11–6·34); aHR: 2·09 (95% CI: 1·25–3·48); and aHR: 2·01 (95% CI: 1·17–3·45)). Among subgroups of male COPD patients without emphysema, with anaemia, without myocardial infarction, without cerebrovascular disease, or with chronic kidney disease, sleep apnoea had a significantly reduced lung cancer hazard (Fig. 2).

Finally, we evaluated whether sleep apnoea treatment changed its impact on lung cancer hazard in (female) patients with COPD. The incident lung cancer hazard remained significantly elevated in COPD females with sleep apnoea using CPAP compared to COPD females without sleep apnoea (aHR: 1·63 (95% CI: 1·11–2·41)). Moreover, in the male population, the hazard was no longer significantly reduced for COPD patients with sleep apnoea using CPAP (aHR: 0·83 (95% CI: 0·66–1·04)). For sleep apnoea patients using oxygen therapy, incident lung cancer hazard remained increased in female COPD patients (aHR: 1·69 (95% CI: 1·17–2·43)) and was no longer significantly reduced for male COPD patients (aHR: 0·99 (95% CI: 0·73–1·36)).

### Sensitivity analyses

During follow-up, 2937 COPD patients received an incident sleep apnoea diagnosis. When these cases were excluded, results remained unchanged in univariable (***χ***-squared univariate: 14·364, P-interaction < 0·001, eFigure S4a) and multivariable analyses (***χ***-squared multivariate: 14·385, P-interaction < 0·001, eFigure S4b). Similarly as in the main analyses, sex differences were especially pronounced in the emphysema, anaemia and cerebrovascular disease subgroups, with sleep apnoea also significantly reducing lung cancer hazards in male COPD patients without chronic kidney disease. (eFigure S4b). The hazard ratio of incident sleep-apnoea on lung cancer, when it is treated as a time-dependent variable was 1·00 (95% CI: 0·69–1·46) for female COPD patients and 0·86 (95% CI: 0·67–1·12) for male COPD patients. The hazard ratio of sleep-apnoea after censoring the first three months, was 1·30 (95% CI: 1·03–1·64) for female COPD patients and 0·85 (95% CI: 0·73–0·99) for male COPD patients. The sex-sleep apnoea interaction also remained significant (***χ***-squared = 13·239, P < 0·001). The observed sex difference remained among different COPD subgroups (eFigure S5). Furthermore, a stratification on smoking status was shown in eFigure S5.

## Discussion

In this Belgian nationwide registration study, a sex-specific impact of sleep apnoea among COPD patients on the risk of lung cancer was observed, indicating a higher risk in the female COPD population, while observing a protective effect among COPD males. This protective effect was not significant for male COPD patients who were treated for sleep apnoea.

This is to our knowledge the first research focussing on the impact of sleep apnoea on lung cancer, specifically in COPD patients. Although the link between sleep apnoea and lung cancer has been studied in the general population, studies were inconclusive.^8^, ^9^, ^10^^,^^12^^,^^13^ However, an increasing number of studies are suggesting a positive association.^8^ Differences between these studies might result from differences in demographics, and morbidities. For example, the study by Huang et al. which observed a sleep apnoea hazard ratio of 1·52 (95% CI: 1·07–2·16) for lung cancer, was based on a female population of median age 73 years.^16^ The study of Jara et al. which observed an aHR of 1·32 (95% CI: 1·27–1·37) was based on veterans, of predominantly male sex (94%).^17^ Finally, the study of Gozal et al., where no significant association was observed (HR: 1·02 (95% CI: 0·99–1·06)), consisted of 50% females aged between 50 and 59 years.^9^ Our results show disparities between sleep apnoea and lung cancer risk by biological sex in those with COPD. This is in line with a recent Korean nationwide cohort, which however did not take COPD into account.^13^ Taking a wide variety of demographic and comorbidity confounders into account, we observed a 31% increased hazard of lung cancer among female COPD patients with sleep apnoea, while we observed an 18% reduced hazard of lung cancer in male COPD patients with sleep apnoea. Previous research has described general sex disparities in sleep apnoea.^14^^,^^18^ Pathophysiological differences have been described including, a lower upper airway collapsibility in females and a prolonged partial upper airway obstruction causing an increased tidal-CO_2_ level.^14^ A greater impairment of life has been reported among women with sleep apnoea compared to men.^18^ Strikingly, women experience more systematic inflammation for a similar apnoea-hypopnoea index,^14^ as levels of fibrinogen and C-reactive protein (CRP) are stronger correlated with sleep apnoea in females. Inflammatory states such as increased CRP levels are associated with an increased lung cancer risk.^8^ Furthermore, women with OSA are shown to have a higher propensity to develop pulmonary hypertension.^15^ These factors could facilitate a higher carcinogenesis predisposition in females with sleep apnoea. In line, a recent study reported a synergetic effect between COPD and OSA, in cardiovascular diseases and mortality, in women but not in men.^19^

The increased hazard ratio of lung cancer was especially pronounced among women with hypoxia-related signs, including anaemia (aHR: 2·09 (95% CI: 1·25–3·48)), cerebrovascular disease (aHR: 2·01 (95% CI: 1·17–3·45)), or emphysema (aHR: 2·65 (95% CI: 1·11–6·34)). Remarkably, female sex, BMI, emphysema and gas trapping have been demonstrated important determinants of the apnoea–hypopnoea index in a subset of the COPDGene study.^20^ However, factors as emphysema are not considered for sleep apnoea generally.^3^ Hypoxia-related comorbidities in COPD could indicate that the increased hazard ratio of lung cancer in female COPD with sleep apnoea is linked to lower diffusion capacity and/or more hypoxia-induced inflammation.^21^ Indeed, sex differences in response to hypoxia have been described.^22^ Furthermore, a recent study confirmed that lower diffusion capacity is associated with a higher apnoea-hypopnoea index and more nocturnal hypoxaemia and desaturation.^23^ Future research could further explain these mechanisms and how these may explain differential underpinnings towards lung cancer.

Our study focuses on women after menopausal age given that the severity of sleep apnoea increases drastically after the menopausal age.^14^ Moreover, women with low serum concentrations of female sex hormones are more likely to suffer from OSA symptoms.^24^ Progesterone is even known to be a respiratory stimulant, which can reduce the frequency of apnoeas.^25^ On the other hand, men with sleep apnoea have less serum testosterone.^26^ Studies have shown an association between testosterone and an increased lung cancer risk.^27^ Moreover, sex-dependent intermittent hypoxia effects have been observed in animal models. Specifically, female rats experienced increased inflammation, with an increased plasma IL6 and IL6/IL10 ratio. This was likely dependent on the amount of mitochondrial oxidative stress.^28^

Furthermore, sleep apnoea is often underdiagnosed and remains undertreated in females compared to males, due to a different clinical presentation.^14^ Studies, focussing specifically on the impact of sex differences in sleep apnoea are needed to fully elucidate the role of differences in hormones, treatment, and underdiagnosis on lung cancer. Noteworthy is the seemingly protective effect of sleep apnoea on lung cancer risk among COPD males. However, on CPAP users and people who received oxygen supplementation, the effect was not significant. This could indicate that there is only a protective effect in less severe sleep apnoea cases. As our study focused on a COPD population to explore hypoxia-related effects, it may reveal different sex-related processes underlying lung cancer, compared to the general population.^6^ When simulating the potential impact of having both OSA and COPD on histological subtypes, conditions mimicking more severe hypoxia enhanced the proliferation of squamous cell carcinoma.^29^ Given that adenocarcinoma are the most common type of primary lung cancer, further research should investigate whether sex differences reflect any shift in specific lung cancer subtypes.^29^

Finally, our study found that the impact of sleep apnoea on lung cancer remained in COPD females, despite the usage of CPAP or oxygen supplementation which could reduce inflammation.^30^ This is probably caused by a more severe underlying sleep apnoea among CPAP users, as also the protective effectiveness in males was no longer significant. Our study lacked information on the adherence of CPAP usage. While CPAP could reduce inflammation, the impact on CRP levels is suggested to be rather mild, therefore more power and further investigation might be required.^30^ The impact of sleep apnoea treatment, accounting for the severity of sleep apnoea, remains an important question for future research. As COPD is a highly prevalent pulmonary disease causing a high predisposition for lung cancer,^6^ it is crucial to unravel underlying biological factors to optimise prevention strategies. Our study indicates a complex relationship between sex differences, intermittent hypoxia, and lung cancer. There is a growing interest in personalised medicine and the usage of low-dose CT scanning for early detection of lung cancer. Female COPD patients with sleep apnoea might be a group that could benefit from closer monitoring and specific interventions against nocturnal hypoxia. Especially as this group has also an increased risk of cardiovascular events.^19^ Future research is essential to provide further insight into the sex-specific effect of sleep apnoea on lung cancer to enhance personalised lung cancer prevention strategies.

Our study had several strengths. While COPD, sleep apnoea and lung cancer have each on their own thoroughly been studied, the interaction between these diseases remains largely unknown. We are to our knowledge the first to describe the impact of sleep apnoea on lung cancer specifically in a COPD population. A major strength is that our cohort consists of a large population of patients with COPD. Furthermore, we adjusted our analyses for a broad range of differential characteristics, comorbidities, and comedication.

Despite these major strengths, we acknowledge several limitations. First, although we controlled for confounders as smoking, residual confounding by smoking intensity or sleep apnoea severity may be present. Second, the observational design makes it difficult to infer causality. However, several mechanisms (including: hypoxic stimulus, hormones, and underdiagnosis) could explain the heterogeneity of the impact of sleep apnoea on lung cancer.

Third, sleep apnoea is a highly prevalent but underdiagnosed condition. This may lead to the misclassification of controls. As milder sleep apnoea cases will more likely remain undiagnosed compared to severe apnoeas, the estimated risk of sleep apnoea might be driven by more severe sleep apnoea cases. As especially women are underdiagnosed for sleep apnoea, it is plausible that more severe cases of sleep apnoea are present among the female COPD population.^3^ However, we adjusted analyses for multiple severity contributors, including comorbidities and comedication.

Fourth, emphysema is often quantified with CT scans, which makes incidental lung cancer diagnosis more likely. However, the whole population consists of hospital-diagnosed COPD patients. Therefore, the whole study population will have a higher medical follow-up. Furthermore, the association was consistent in other hypoxia-related subgroups not defined by CT and the increase in lung cancer diagnosis was proportional over time.

Fifth, the look-back period for defining comorbidities and comedication is one-year except for smoking. Therefore, we might miss older registered comorbidities or cancer history.

In conclusion, with this nationwide cohort study the specific impact of sleep apnoea on lung cancer risk is investigated, in a COPD population. Our results illustrate major sex discrepancies in the effect of sleep apnoea on lung cancer. While sleep apnoea was associated with a higher risk of lung cancer among females, data revealed a lower risk among males suffering from sleep apnoea. The sex differences were especially evident among females with hypoxia-related comorbidities.

## Contributors

KP and LL had direct access to the raw data and verified the reported data. KP and LL contributed to the investigation, conceptualisation, the methodology of the study. KP provided the data curation, performed the formal analyses and visualisation, and wrote the original draft under the supervision of LL. LL and AL critically revised the manuscript and edited it. LL contributed to the project administration and funding acquisition and made the final decision for submission. All authors contributed to the article and approved the final manuscript.

## Data sharing statement

Requests for the individual participant data, after de-identification, underlying this article should be directed to the administrators of the InterMutualistic Agency (IMA) database or Minimal Hospital Dataset and are subject to approval.

## Declaration of interests

Outside this manuscript, LL has been consulted as expert for AstraZeneca, GlaxoSmithKline and Sanofi, and has given lectures sponsored by IPSA vzw and Domus Medica vzw (non-profit organisations facilitating lifelong learning for health care providers), Chiesi, Johnson and Johnson, all paid to her institution. She received support for travel from Menarini. None of which are related to the content of this work. Furthermore, LL is a member of the American Thoracic Society, European Respiratory Society and Belgian Respiratory Society, her Faculty board and faculty committees. The authors declare that the research was conducted in the absence of any commercial or financial relationships that could be construed as a potential conflict of interest.
