# Supplementary material for: Sex-specific associations between sleep apnoea and lung cancer risk in patients with COPD: a nationwide prospective cohort study
Source: Lancet Reg Health Eur. 2025 Mar 20;52:101269. doi: 10.1016/j.lanepe.2025.101269 (PMC11987683; doi:10.1016/j.lanepe.2025.101269)
Supplement: Supplementary Materials [file mmc1.docx]

# **Supplemental tables**

## **eTable1: Compliance to the STROBE (Strengthening the Reporting of Observational Studies in Epidemiology) reporting guideline.**^1^

|  | Item No. | Recommendation | Page No. |
| --- | --- | --- | --- |
| *Title and abstract* | 1 | (*a*) Indicate the study’s design with a commonly used term in the title or the abstract | 1-2 |
|  |  | (*b*) Provide in the abstract an informative and balanced summary of what was done and what was found | 2 |
| *Introduction* | | |  |
| Background/rationale | 2 | Explain the scientific background and rationale for the investigation being reported | 4-5 |
| Objectives | 3 | State specific objectives, including any prespecified hypotheses | 5 |
| *Methods* | | |  |
| Study design | 4 | Present key elements of study design early in the paper | 4-6 |
| Setting | 5 | Describe the setting, locations, and relevant dates, including periods of recruitment, exposure, follow-up, and data collection | 4-6 |
| Participants | 6 | (*a*) Give the eligibility criteria, and the sources and methods of selection of participants. Describe methods of follow-up | 5-6 |
|  |  | (*b*) For matched studies, give matching criteria and number of exposed and unexposed | 5-6 |
| Variables | 7 | Clearly define all outcomes, exposures, predictors, potential confounders, and effect modifiers. Give diagnostic criteria, if applicable | 5-8, eTable 2 |
| Data sources/ measurement | 8* | For each variable of interest, give sources of data and details of methods of assessment (measurement). Describe comparability of assessment methods if there is more than one group | 5-8 eTable 2 |
| Bias | 9 | Describe any efforts to address potential sources of bias | 5-8 |
| Study size | 10 | Explain how the study size was arrived at | 5-8 |
| Quantitative variables | 11 | Explain how quantitative variables were handled in the analyses. If applicable, describe which groupings were chosen and why | 5-8 |
| Statistical methods | 12 | (*a*) Describe all statistical methods, including those used to control for confounding | 6-8 |
|  |  | (*b*) Describe any methods used to examine subgroups and interactions | 6-8 |
|  |  | (*c*) Explain how missing data were addressed | / |
|  |  | (*d*) If applicable, explain how loss to follow-up was addressed | / |
|  |  | (*e*) Describe any sensitivity analyses | 8 |
| *Results* | | |  |
| Participants | 13* | (a) Report numbers of individuals at each stage of study—eg numbers potentially eligible, examined for eligibility, confirmed eligible, included in the study, completing follow-up, and analysed | 8-9, Table 1, eTable 1 |
|  |  | (b) Give reasons for non-participation at each stage | / |
|  |  | (c) Consider use of a flow diagram | / |
| Descriptive data | 14* | (a) Give characteristics of study participants (eg demographic, clinical, social) and information on exposures and potential confounders | Table 1, eTable 1 |
|  |  | (b) Indicate number of participants with missing data for each variable of interest | Table 1 |
|  |  | (c) Summarise follow-up time (eg, average and total amount) | 7, Table 1, eTable 2 |
| Outcome data | 15* | Report numbers of outcome events or summary measures over time | 7-10, Table 1, |
| Main results | 16 | (*a*) Give unadjusted estimates and, if applicable, confounder-adjusted estimates and their precision (eg, 95% confidence interval). Make clear which confounders were adjusted for and why they were included | 7-13, Figure 1-2, efigures 2-3 |
|  |  | (*b*) Report category boundaries when continuous variables were categorized | Table 1 |
|  |  | (*c*) If relevant, consider translating estimates of relative risk into absolute risk for a meaningful time period | / |
| Other analyses | 17 | Report other analyses done—eg analyses of subgroups and interactions, and sensitivity analyses | Figure 1-2, efigures 2-3 |
| *Discussion* | | |  |
| Key results | 18 | Summarise key results with reference to study objectives | 13-15 |
| Limitations | 19 | Discuss limitations of the study, taking into account sources of potential bias or imprecision. Discuss both direction and magnitude of any potential bias | 15-17 |
| Interpretation | 20 | Give a cautious overall interpretation of results considering objectives, limitations, multiplicity of analyses, results from similar studies, and other relevant evidence | 15-17 |
| Generalisability | 21 | Discuss the generalisability (external validity) of the study results | 15-17 |
| *Other information* | | |  |
| Funding | 22 | Give the source of funding and the role of the funders for the present study and, if applicable, for the original study on which the present article is based | 8,18 |

**Give information separately for exposed and unexposed groups.*

## **eTable2: Definition of in- and exclusion criteria, comorbidities, medication history and clinical risk scores**

| **Outcome variables** | **ICD, ATC, AND MEDICAL PROCEDURE CODES** |
| --- | --- |
| **Exclusion criteria** |  |
| Tumoral lesion | *ICD-10:*  C00-C96, Z510, Z5111, Z5112  *ATC:*  L01  *Medical procedure code:*  154873, 154884, 154895, 154906, 157231, 157242, 201191, 201202, 201213, 201224, 220275, 220286, 220371, 220382, 201213, 201224, 226914, 226925, 226936, 226940, 227216, 227220, 227275, 227286, 227636, 227640, 227651, 227662, 227673, 227684, 227695, 227706, 227710, 227721, 227732, 227743, 227754, 227765, 227776, 227780, 227791, 227802, 227813, 227824, 227835, 227846, 228012, 228023, 228174, 228185, 228233, 228244, 228255, 228266, 228270, 228281, 228292, 228303, 228314, 228325, 228336, 228340, 230473, 230484, 231033, 231044, 241231, 241242, 241415, 241426, 241430, 241441, 241452, 241463, 241555, 241566, 242012, 242023, 242034, 242045, 242292, 242303, 242314, 242325, 242830, 242841, 242852, 242863, 242874, 242885, 242896, 242900, 243051, 243062, 243073, 243084, 243235, 243246, 243736, 243740, 243751, 243762, 243773, 243784, 244016, 244020, 244031, 244042, 244075, 244086, 244790, 244801, 244856, 244860, 244893, 244904, 244915, 244926, 244930, 244941, 244952, 244963, 244974, 244985, 245512, 245523, 245534, 245545, 246050, 246061, 246072, 246083, 247111, 247122, 247133, 247144, 251753, 251764, 251775, 251786, 254892, 254903, 256115, 256126, 256336, 256340, 256572, 256583, 257191, 257202, 258355, 258366, 258370, 258381, 258392, 258403, 258451, 258462, 258554, 258565, 258856, 258860, 258871, 258882, 258893, 258904, 259033, 259044, 259114, 259125, 260190, 260201, 260411, 260422, 260433, 260444, 260551, 260562, 260654, 260665, 260750, 260761, 261111, 261122, 261391, 261402, 261472, 261483, 261671, 261682, 261774, 261785, 261796, 261800, 262334, 262345, 262570, 262581, 277756, 277760, 277771, 277782, 278795, 278806, 278810, 278821, 281831, 281842, 281956, 281960, 282310, 282321, 282671, 282682, 284056, 284060, 288455, 288466, 288470, 288481, 289892, 289903, 291056, 291060, 310494, 310505, 311312, 311323, 312550, 312561, 312572, 312583, 312594, 312605, 312653, 312664, 312970, 312981, 350114, 350125, 350136, 350140, 350276, 350280, 350291, 350302, 350372, 350383, 350674, 350685, 350696, 350700, 431174, 431185, 431336, 431340, 431351, 431362, 432294, 432305, 444113, 444124, 444135, 444146, 444150, 444161, 444172, 444183, 444194, 444205, 444216, 444220, 444231, 444242, 444253, 444264, 444275, 444286, 444290, 444301, 444312, 444323, 444334, 444345, 444474, 444485, 444592, 444603, 473970, 473981, 474795, 474806, 565073, 565084, 565095, 565106, 565110, 565121, 565132, 565143, 565154, 565165, 587834, 587845, 587871, 587882, 587893, 587904, 587915, 587926, 588431, 588442, 588453, 588464, 588475, 588486, 588490, 588501, 588512, 588523, 588534, 588545, 588556, 588560, 588571, 588582, 588593, 588604, 588770, 588781, 588976, 588980, 589691, 589702, 589713, 589724, 589831, 589842, 589875, 589886, 594016, 594020, 594031, 594042, 594053, 594064, 594075, 594086, 594090, 594101, 594112, 594123, 594252, 594263, 594274, 594285, 594296, 594300, 594311, 594322, 594333, 594344, 594355, 594366, 594370, 594381, 594392, 594403, 594414, 594425, 594436, 594440, 594451, 594462, 594495, 594506, 594510, 594521, 594532, 594543, 594554, 594565, 594576, 594580, 594591, 594602, 594613, 594624, 594635, 594646, 594694, 594705, 594716, 594720, 594753, 594764, 594775, 594786, 594790, 594801, 594812, 594823, 594834, 594845, 594856, 594860, 594871, 594882, 594893, 594904, 594915, 594926, 594930, 594941, 598581, 682636, 682640, 682732, 682743, 687934, 687945, 698051, 698062, 698095, 698106, 698390, 698401, 698456, 698460, 698471, 698482, 698493, 698504, 698530, 698541, 745010, 745021, 745032, 745043, 745113, 745124, 745135, 745146, 745150, 745161 |
| Chronic obstructive pulmonary disease | *ICD-10:*  J40-J44 |
| **Exposure variables** |  |
| Sleep apnoea | *ICD-10:*  G47·3  *Medical procedure code:*  765951,  Personal contribution of the patient to ventilation at home through continuous pressure over the nose (nCPAP) during sleep  779870, 779881,  MRA starting lump sum for a new MRA patient  779892, 779903,  MRA starting lump sum in case of renewal of the MRA  779914, 779925,  MRA basic lump sum  779936,  nCPAP starting lump sum  779951,  nCPAP basic lump sum  788012, 788023  Nocturnal respiratory support via continuous positive pressure at two levels for SOH patients |
| Oxygen | *ATC:*  V03AN01  *Medical procedure code:*  355095, 355106, 355110, 355121, 523795, 523806, 751030, 751052, 751236, 754132, 754493, 754574, 755370, 755952, 757455, 757470, 757831, 760642, 797252, 797274, 797296, 797311, 797333, 797355, 797370, 797392 |
| CPAP | *Medical procedure code:*  765951, 779936, 779951 |
| **Outcome variables** |  |
| Lung cancer | *ICD-10:*  C34, C384, C450, C780, C782  *Medical procedure code:*  227216, 227220  Extensive total or partial lung exeresis with glandular evidence for oncological disease  594311, 594322,  Detecting an activating EGFR mutation in primary advanced (unresectable or metastatic) non-squamous non-small cell lung carcinoma  594333, 594344,  Analysis of ALK gene rearrangement in ALK positive (IHC) advanced (unresectable or metastatic) non-squamous non-small cell lung carcinoma  594355, 594366,  Analysis of ROS1 gene rearrangement in ROS1 positive (IHC) advanced (unresectable or metastatic) non-squamous non-small cell lung carcinoma  594370, 594381  Detection of EGFR T790M mutation during progression during or after treatment with an EGFR TKI of advanced (unresectable or metastatic) non-squamous non-small cell lung carcinoma |
| **Comorbidities** |  |
| Alcoholism | *ICD-10:*  E244, E51, F10, G312, G621, G721, I426, K292, K70, K852, K860, O354, T51, Z714  *Medical procedure code:*  790090, 790473, 791335, 791431 |
| Anemia | *ICD-10:*  D460, D461, D462, D50, D51, D52, D53, D55, D56, D57, D58, D59, D60, D61, D62, D63, D64 |
| Asthma | *ICD-10:*  J45 |
| Cerebrovascular disease | *ICD-10:*  G45, G46, H340, I60, I61, I62, I63, I65, I66, I67, I68, I69, Z8673  *Medical procedure code:*  182136, 182140, 182151, 182162, 182173, 182184, 477724, 477746, 477761, 477783 |
| Chronic kidney disease | *ICD-10:*  107096, 107111, 107133, 107155, 318010, 318021, 318290, 318301, 470293, 470304, 470315, 470326, 470330, 470341, 470352, 470374, 470385, 470400, 470422, 470433, 470444, 470466, 470470, 470481, 470875, 470890, 470901, 470912, 470934, 470945, 471111, 471122, 471133, 471144, 471155, 471166, 471170, 471181, 474714, 474725, 754294, 757433, 757492, 761272, 761283, 761456, 761471, 761493, 761515, 761526, 761530, 761552, 761574, 761596, 761655, 761I120, I1311, I132, N032, N033, N034, N035, N036, N037, N052, N053, N054, N055, N056, N057, N18, N19, N250, T861, Z4822, Z49, Z9115, Z940, Z992  *Medical procedure code:*  107096, 107111, 107133, 107155, 318010, 318021, 318290, 318301, 470293, 470304, 470315, 470326, 470330, 470341, 470352, 470374, 470385, 470400, 470422, 470433, 470444, 470466, 470470, 470481, 470875, 470890, 470901, 470912, 470934, 470945, 471111, 471122, 471133, 471144, 471155, 471166, 471170, 471181, 474714, 474725, 754294, 757433, 757492, 761272, 761283, 761456, 761471, 761493, 761515, 761526, 761530, 761552, 761574, 761596, 761655, 761670, 767594, 767616, 767631, 767664, 767686, 767701, 767723, 767734, 767756, 767782, 767804, 767815, 767826, 767830, 767841, 767955, 767966 |
| Diabetes Mellitus | *ICD-10:*  E08, E09, E10, E11, E13, E14, Z4681, Z9641  *ATC:*  A10  *Medical procedure code:*  102852, 107015, 107030, 107052, 107074, 109594, 174370, 174381, 174392, 174403, 174414, 174425, 174436, 174440, 174451, 174462, 174473, 174484, 174495, 174506, 174510, 174521, 423135, 423150, 423172, 423194, 423216, 423231, 423334, 423813, 423835, 423850, 433554, 433565, 540772, 540783, 543712, 543723, 653671, 653682, 697093, 697104, 754176, 754191, 754250, 754272, 754736, 757352, 757374, 757396, 757411, 757514, 757536, 757551, 757573, 770033, 770055, 770070, 771573, 771595, 773393, 773496, 784630, 784641, 784652, 784663, 785735, 785750, 785772, 785794, 785816, 785831, 785853, 785875, 785890, 785912, 785934, 785956, 786015, 786030, 786100, 788756, 788771, 788793, 788815, 788830, 788852, 788874, 788896, 788911, 788933, 788955, 789751, 789773, 789795, 789810, 789832, 789854, 789876, 789891, 789913, 789935, 794032, 794054, 794076, 794091, 794253, 794275, 794290, 794312, 794334, 794356, 794371, 794393, 794415, 794430, 794452, 961295, 961306, 961332, 961343 |
| Emphysema | *ICD 10:*  J43, J982, J983 |
| Hypertension | *ICD-10:*  I10-I13, I15, I16, I67·4  *ATC:*  Combination treatment with ≥2 of the following drug classes:   - Cardioselective beta blocker: C07AB, C07AG, C07BB, C07BG, C07CB, C07CG, C07DB, C07FB, C07FX03, C07FX04, C07FX05, C07FX06, C09BX02, C09BX04, C09BX05, C09DX05 - ACE inhibitor or angiotensin II receptor blocker: C09A, C09B, C09C, C09D, C10BX04, C10BX06, C10BX07, C10BX10, C10BX11, C10BX12, C10BX13, C10BX14, C10BX15, C10BX16, C10BX17, C10BX18 - Calcium channel blocker: C07FB, C08C, C08G, C09BB, C09DB, C09BX01, C09BX03, C09BX04, C09DX01, C09DX03, C09DX06, C09DX07, C09XA53, C09XA54, C10BX03, C10BX07, C10BX09, C10BX11, C10BX14, C10BX18 - Non-loop diuretic: C02L, C03A, C03BA, C03BB, C03EA, C07B, C07C, C07D, C08GA, C09BA, C09BX01, C09BX03, C09DA, C09DX01, C09DX03, C09DX06, C09DX07, C09XA52, C09XA54, C10BX13, C03D, C03EA, C03EB   Other antihypertensive (alpha adrenergic blocker, vasodilator): C02A, C02B, C02C, C02DB, C02DD, C02DG, C02L |
| Myocardial infarction | *ICD-10:*  I21, I22, I252 |
| Obesity or overweight | *ICD-10:*  E66, Z68·25-Z68·29, Z68·3, Z68·4  *ATC:*  A08AA62, A08AB01  *Medical procedure code:*  241776, 241780, 241813, 241824, 241835, 241846 |
| Peripheral vascular disease | *ICD-10:*  I70, I71, I73·1, I73·8, I73·9, I74, I77, I79·0, K55·1, K55·8, K55·9, Z95·82, Z98·62  *Medical procedure code:*  229294, 229305, 229316, 229320, 229331, 229342, 235071, 235082, 235093, 235104, 235115, 235126, 235196, 235200, 235211, 235222, 236014, 236025, 236036, 236040, 236051, 236062, 237016, 237020, 237031, 237042, 237053, 237064, 237075, 237086, 237090, 237101, 237171, 237182, 589050, 589061, 589094, 589105, 589175, 589186, 589595, 589606, 589610, 589621, 589632, 589643, 589654, 589665 |
| Pneumonia | *ICD-10:*  A0103, A0222, A3701, A3711, A3781, A3791, A5004, A5484, B012, B052, B0681, B7781, J09X1, J09X2, J09X3, J100, J110, J12, J13, J14, J15, J16, J17, J18, J8411, J842, J851, J95851, Z8701 |
| Socioeconomic status | Dummy variable derived from medical coverage. This is based on copayments for medication or medical procedures at the index date. |
| **Medication history** |  |
| Inhaled corticosteroids | *ATC:*  R03AK06, R03AK07, R03AK08, R03AK10, R03AK11, R03AK12,R03AK14, R03AL08, R03AL09, R03AL11, R03AL12, R03BA01, R03BA02, R03BA05 |
| Sedatives and hypnotic drugs | *ATC:*  N05C |
| **Clinical risk scores (at baseline)** |  |
| Charlson Comorbidity Index² | 1. Myocardial infarction: 1 point:    - *ICD-10:* I21, I22 2. Congestive heart failure: 1 point:  - *ICD 10:* I09·81, I11·0, I13·0, I13·2, I42·0, I42·6-I42·9, I43, I50 - *ATC:* combination treatment of the following classes of drugs:   - Cardioselective beta blockers: C07AB, C07AG, C07BB, C07BG, C07CB, C07CG, C07DB, C07FB, C07FX03, C07FX04, C07FX05, C07FX06, C09BX02, C09BX04, C09BX05, C09DX05   - ACE inhibitors or angiotensin II receptor blockers: C09A, C09B, C09C, C09D, C10BX04, C10BX06, C10BX07, C10BX10, C10BX11, C10BX12, C10BX13, C10BX14, C10BX15, C10BX16, C10BX17, C10BX18   - Potassium-sparing diuretics: C03D, C03EA, C03EB   - Loop diuretics: C03C, C03EB  1. Peripheral vascular disease: 1 point (definition mentioned above: ‘Peripheral artery disease’) 2. Cerebrovascular disease: 1 point  - *ICD-10:* I61, I62, I63, I65, I66, I67, I68, I69, G45, G46, H34·0  1. Dementia: 1 point  - *ICD-10:* A81·0, F01, F02, F03, F10·27, F10·97, G30, G31·0, G31·83, G31·85 - *ATC:* N06D  1. Connective tissue disease: 1 point:  - *ICD-10:* D69·0, M30, M31, M32, M33, M34, M35, M36·0, M36·8  1. Peptic ulcer disease: 1 point:  - *ICD-10:* B96·81, K22·1, K25, K26, K27, K28, Z87·11 - *ATC:* A02BD04, A02BD11 - *Medical procedure code:* 550093, 550104, 552370, 552381  1. Mild liver disease: 1 point:  - *ICD-10:* B17·0, B17·10, B18, B19·10, B19·20, K70·0, K70·1, K70·2, K70·3, K70·9, K71·3, K71·4, K71·5, K71·6, K71·7, K71·8, K71·9, K73, K74, K75·3, K75·4, K75·8, K75·9, K76·0, K76·1, K76·2, K76·89, K76·9, K77 - *ATC:* J05AB04, J05AF05, J05AF07, J05AF08, J05AF10, J05AE11, J05AE12, J05AE14, J05AX15, J05AX65, J05AP - *Medical procedure code:* 556754, 556765  1. Diabetes without chronic complications: 1 point  - *ICD-10:* E08·2-E08·5, E08·8, E09·2-E09·5, E09·8, E10·2-E10·5, E10·8, E11·2-E11·5, E11·8, E13·2-E13·5, E13·8 - *Medical procedure code:* 653671, 653682, 697093, 697104, 770070, 773393, 773496  1. Diabetes with chronic complications: 2 points  - *ICD-10:* E08·0, E08·1, E08·6, E08·9, E09·0, E09·1, E09·6, E09·9, E10·1, E10·6, E10·9, E11·0, E11·1, E11·6, E11·9, E13·0, E13·1, E13·6, E13·9  1. Hemiplegia or paraplegia: 2 points:  - *ICD-10:* G04·1, G11·4, G80·0, G80·1, G80·2, G81, G82, G83·0, G86·9, I69·05, I69·15, I69·25, I69·35, I69·85, I69·95 - *Medical procedure code:* 643414, 643425  1. Renal disease: 2 points  - *ICD-10:* N18·5, N18·6, N19, I12·0, I13·11, T86·1, Z49, Z94·0, Z99·2 - *Medical procedure code:* N81 (group code), 318010, 318021, 318290, 318301  1. Any malignancy, including leukemia and lymphoma: 2 points:  - *ICD-10:* C00-C76, C80·1, C80·2, C81-C96, D00-D09, D37-D49, E31·2, Z51·0, Z51·11, Z51·12 - *ATC:* L01 - *Medical procedure code:* 154873, 154884, 154895, 154906, 157231, 157242, 201191, 201202, 201213, 201224, 220275, 220286, 220371, 220382, 201213, 201224, 226914, 226925, 226936, 226940, 227216, 227220, 227275, 227286, 227636, 227640, 227651, 227662, 227673, 227684, 227695, 227706, 227710, 227721, 227732, 227743, 227754, 227765, 227776, 227780, 227791, 227802, 227813, 227824, 227835, 227846, 228012, 228023, 228174, 228185, 228233, 228244, 228255, 228266, 228270, 228281, 228292, 228303, 228314, 228325, 228336, 228340, 230473, 230484, 231033, 231044, 241231, 241242, 241415, 241426, 241430, 241441, 241452, 241463, 241555, 241566, 242012, 242023, 242034, 242045, 242292, 242303, 242314, 242325, 242830, 242841, 242852, 242863, 242874, 242885, 242896, 242900, 243051, 243062, 243073, 243084, 243235, 243246, 243736, 243740, 243751, 243762, 243773, 243784, 244016, 244020, 244031, 244042, 244075, 244086, 244790, 244801, 244856, 244860, 244893, 244904, 244915, 244926, 244930, 244941, 244952, 244963, 244974, 244985, 245512, 245523, 245534, 245545, 246050, 246061, 246072, 246083, 247111, 247122, 247133, 247144, 251753, 251764, 251775, 251786, 254892, 254903, 256115, 256126, 256336, 256340, 256572, 256583, 257191, 257202, 258355, 258366, 258370, 258381, 258392, 258403, 258451, 258462, 258554, 258565, 258856, 258860, 258871, 258882, 258893, 258904, 259033, 259044, 259114, 259125, 260190, 260201, 260411, 260422, 260433, 260444, 260551, 260562, 260654, 260665, 260750, 260761, 261111, 261122, 261391, 261402, 261472, 261483, 261671, 261682, 261774, 261785, 261796, 261800, 262334, 262345, 262570, 262581, 277756, 277760, 277771, 277782, 278795, 278806, 278810, 278821, 281831, 281842, 281956, 281960, 282310, 282321, 282671, 282682, 284056, 284060, 288455, 288466, 288470, 288481, 289892, 289903, 291056, 291060, 310494, 310505, 311312, 311323, 312550, 312561, 312572, 312583, 312594, 312605, 312653, 312664, 312970, 312981, 350114, 350125, 350136, 350140, 350276, 350280, 350291, 350302, 350372, 350383, 350674, 350685, 350696, 350700, 431174, 431185, 431336, 431340, 431351, 431362, 432294, 432305, 444113, 444124, 444135, 444146, 444150, 444161, 444172, 444183, 444194, 444205, 444216, 444220, 444231, 444242, 444253, 444264, 444275, 444286, 444290, 444301, 444312, 444323, 444334, 444345, 444474, 444485, 444592, 444603, 473970, 473981, 474795, 474806, 565073, 565084, 565095, 565106, 565110, 565121, 565132, 565143, 565154, 565165, 587834, 587845, 587871, 587882, 587893, 587904, 587915, 587926, 588431, 588442, 588453, 588464, 588475, 588486, 588490, 588501, 588512, 588523, 588534, 588545, 588556, 588560, 588571, 588582, 588593, 588604, 588770, 588781, 588976, 588980, 589691, 589702, 589713, 589724, 589831, 589842, 589875, 589886, 594016, 594020, 594031, 594042, 594053, 594064, 594075, 594086, 594090, 594101, 594112, 594123, 594252, 594263, 594274, 594285, 594296, 594300, 594311, 594322, 594333, 594344, 594355, 594366, 594370, 594381, 594392, 594403, 594414, 594425, 594436, 594440, 594451, 594462, 594495, 594506, 594510, 594521, 594532, 594543, 594554, 594565, 594576, 594580, 594591, 594602, 594613, 594624, 594635, 594646, 594694, 594705, 594716, 594720, 594753, 594764, 594775, 594786, 594790, 594801, 594812, 594823, 594834, 594845, 594856, 594860, 594871, 594882, 594893, 594904, 594915, 594926, 594930, 594941, 598581, 682636, 682640, 682732, 682743, 687934, 687945, 698051, 698062, 698095, 698106, 698390, 698401, 698456, 698460, 698471, 698482, 698493, 698504, 698530, 698541, 745010, 745021, 745032, 745043, 745113, 745124, 745135, 745146, 745150, 745161  1. Moderate or severe liver disease: 3 points  - *ICD-10:* B15·0, B16·0, B16·2, B17·11, B19·0, B19·11, B19·21, C22, C78·7, I85, I86·4, K65·2, K70·2, K70·3, K70·4, K71·1, K71·7, K72, K74, K76·1, K76·5, K76·6, K76·7, K76·81, R18·8, Z94·4 - *Medical procedure code:* 318076, 318080, 318334, 318345, 472113, 472124, 589352, 589363  1. Metastatic solid tumor: 6 points  - *ICD-10:* C77-C79, C80·0  1. AIDS/HIV: 6 points  - *ICD-10:* B20, Z21 - ATC: J05AE01, J05AE02, J05AE03, J05AE04, J05AE05, J05AE07, J05AE08, J05AE09, J05AE10, J05AF01, J05AF02, J05AF03, J05AF04, J05AF05, J05AF06, J05AF09, J05AF11, J05AF12, J05AF13, J05AG, J05AR, J05AX07, J05AX08, J05AX09, J05AX12  1. Age:   <50 years: 0 points  50-59 years: 1 point  60-69 years: 2 points  70-79 years: 3 points  ≥80 years: 4 points  *The following comorbid conditions were mutually exclusive: diabetes with chronic complications and diabetes without chronic complications; mild liver disease and moderate or severe liver disease; and any malignancy and metastatic solid tumor.* |
| John Hopkins Claims-based Frailty Indicator³ | 1. *Impaired mobility:* beta coefficient 1·24:    - *ICD-10:* G11, G32·81, M62·3, R26, R29·6, Z74·01, Z74·09, Z99·3    - *Medical procedure code:* N83, 643451, 643462, 653656, 653660, 770394, 770405, 770416, 770420 2. *Depression:* beta coefficient 0·54:    - *ICD-10:* F06·31, F06·32, F30, F31, F32, F33, F34·1, F43·21, F43·23    - *ATC:* N06A 3. *Congestive heart failure:* beta coefficient 0·50: (definition mentioned above: ‘Congestive heart failure’) 4. *Parkinson’s disease:* beta coefficient 0·50: (definition mentioned above: ‘Parkinson’s disease’) 5. *White race:* beta coefficient -0·49: not available 6. *Arthritis (any type):* beta coefficient 0·43:    - *ICD-10:* L40·5, M02·1, M02·3, M05, M06, M07, M08, M13·0, M13·1, M45, M46·1, M46·8, M46·9, Z87·39    - *ATC:* L04AA13, L04AA24, L04AA29, L04AA37    - *Medical procedure code:* 478030, 478041 7. *Cognitive impairment:* beta coefficient 0·33:    - *ICD-10:* G31·1, G31·84, G31·89, G31·9, R41·81 8. *Charlson comorbidity index (> 0):* beta coefficient 0·31 9. *Stroke:* beta coefficient 0·28: (definition mentioned above: ‘Stroke’) 10. *Paranoia:* beta coefficient 0·24:     - *ICD-10:* F06·0, F06·2, F20, F22, F23, F24, F28, F29 11. *Chronic skin ulcer:* beta coefficient 0·23:     - *ICD-10:* E08·621, E08·622, E09·621, E09·622, E10·621, E10·622, E11·621, E11·622, E13·621, E13·622, L89, L97, L98·4     - *Medical procedure code:* 114074, 114085 12. *Pneumonia*: beta coefficient 0·21:     - *ICD-10:* A48·1, J11·0, J12, J13, J14, J15, J16, J17, J18 13. *Male sex:* beta coefficient -0·19 14. *Skin and soft tissue infection:* beta coefficient 0·18:     - *ICD-10:* L00, L01, L02, L03, L04, L05, L08 15. *Mycoses:* beta coefficient 0·14:     - *ICD-10:* B35, B36, B37, B38, B39, B40, B41, B42, B43, B44, B45, B46, B47, B48, B49 16. *Age (in 5 year categories):* beta coefficient 0·09 17. *Admission in past 6 months:* beta coefficient 0·09 18. *Gout or other crystal-induced arthropathy:* beta coefficient 0·08:     - *ICD-10:* M10, M11, M1A     - *ATC:* M04A 19. *Falls:* beta coefficient 0·08: (definition mentioned above: ‘History of falls’) 20. *Musculoskeletal problems:* beta coefficient 0·05:     - *ICD-10:* M07, M12·0, M12·1, M12·2, M12·3, M12·4, M12·8, M12·9, M13, M14, M24·0, M24·3, M24·6, M24·7, M24·8, M24·9, M25, M45, M46·0, M46·1, M46·4, M46·8, M46·9, M47-M51, M53, M54, M80, M81, M84·3, M84·4, M84·5, M84·6, Z87·31 21. *Urinary tract infection:* beta coefficient 0·05:     - *ICD-10:* N30·0, N30·8, N30·9, N10, N12, N13·6, N15·9, N16, N34, N39·0     - *ATC****:*** J01XE01, J01XX01 |
| **Smoking status** |  |
| Smoking status | From January 1st, 2010 till follow-up:  *ICD-9:* 305·1, V15·82  *ICD-10:* F17·2, Z71·6, Z72·0, Z87·891  Based on the last registered ICD code, prior to follow-up. Smokers were divided as current smoker and past smoker   - current smoking ICD: ICD9: 305·1 or ICD-10: F17·200, F17·203, F17·208, F17·209, F17·210, F17·213, F17·218, F17·219, F17·220, F17·228, F17·290, F17·293, F17·298, F17·299,Z71·6, Z72·0 - past smoking ICD: history of tobacco use ~ ICD-9: V15·82 or ICD-10: F17·201, F17·211, F17·221, F17·291, Z87·891   In case multiple ICD codes were registered on the same day, current smoking ICD codes were prioritized over past smoking.  Based on the time between the last registration of tobacco use and the index date, ever smokers were considered current smokers if a cessation attempt was performed after follow-up or considered past smoker in case of any smoking cessation attempt before follow-up (see below). |
| Smoking cessation attempt | *ATC:* N06AX12, N07BA  *Medical procedure code:* 740434, 740445, 740456, 740460, 740471, 740482 |

Definition of in- and exclusion criteria, comorbidities related to lung cancer or sleep apnoea,^2–8^ medication history and clinical risk scores based on ICD-coded hospital discharge diagnoses (ICD-9-CM up to 2014 and ICD-10-BE from 2015 onward${s,}^{4}$ medical procedure code$s^{5}$ and/or ATC-coded prescription claim${s.}^{6}$

AF: Atrial fibrillation; ATC: Anatomical Therapeutic Chemical Classification; CKD: Chronic kidney disease; ICD-9-CM: International Classification of Diseases (ICD) codes, 9th revision, Clinical Modification; ICD-10-BE: International Classification of Diseases (ICD) codes, 10th Revision, Belgian Modification.

# **Supplemental figures**

## **eFigure1: Overview of study design**

**
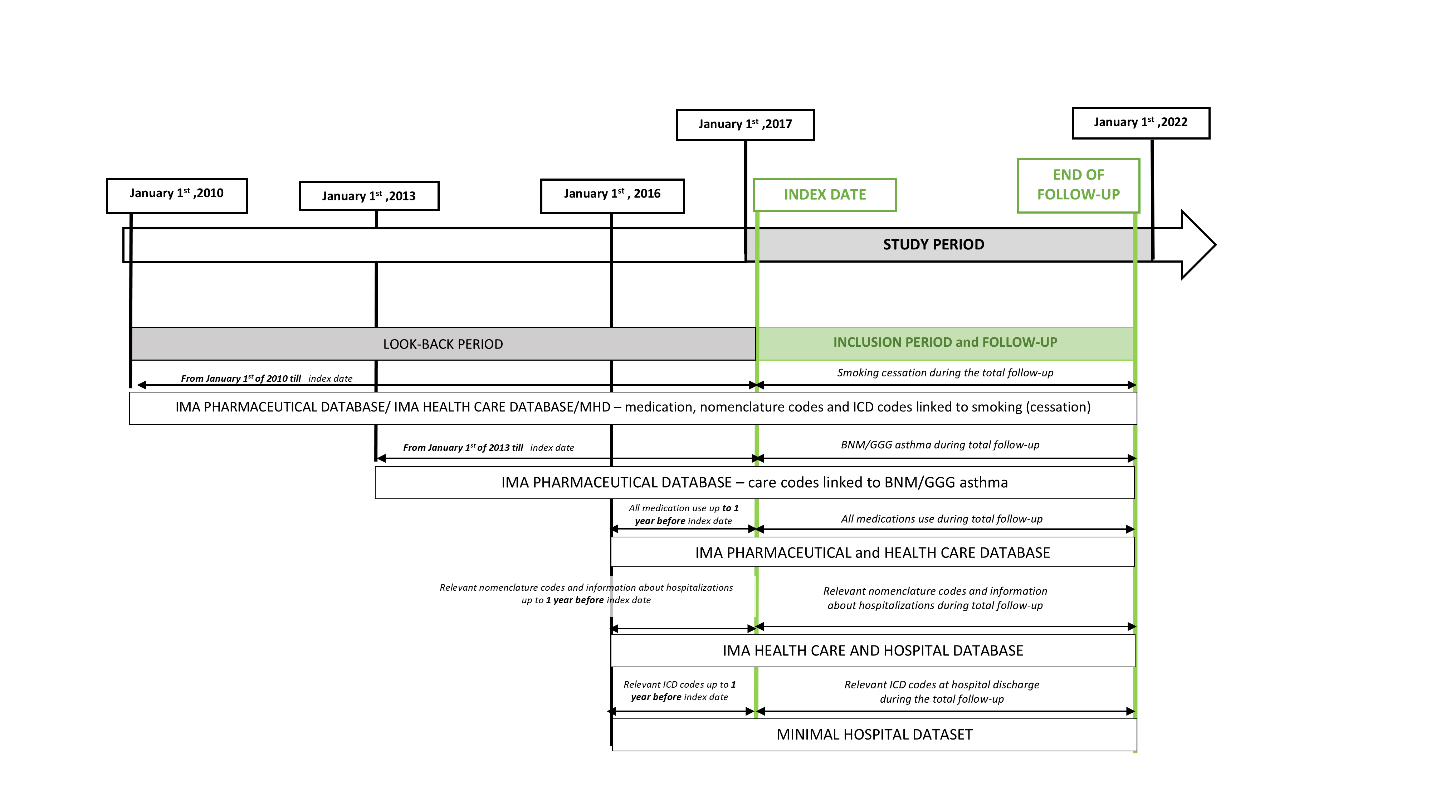
**

*Study period:* January 1st, 2017 – January 1st, 2022. *Index date:* Second filled prescription of R03 medication within the same year (starting from January 1st, 2017), *Look-back period:* One year look back period from the index date for pharmaceutical and health care. For smoking cessation tools and smoking ICD-codes the look-back period ranged back up until January 1^st^ 2010***.*** *End of follow-up:* Patients were followed from the index date (second R03 prescription in one year) till the first occurrence of the investigated outcome, an incident diagnosis of lung cancer, death, emigration, or end of the study period (January 1st, 2019). *Exclusion criteria:* (1) subjects had a COPD diagnosis, therefore people without a COPD-related ICD code (J40-J44) at baseline were excluded. (2) People with a diagnosis related to a tumoral lesion within one year before index date were exlcuded. (3) People younger than 55 years were excluded. *Comorbidities:* Comorbidities were identified using specific ICD-coded diagnoses (e.g. tumoral lesion) from the MHD, medical procedure codes (e.g. cancer-related surgery) from the IMA database and/or ATC-coded prescription claims (e.g. antineoplastic drugs) from the IMA database ≤1 year before the index date. *Comedication use:* Comedication dispensed up to the one-year look-back period.

**eFigure 2:** **Direct acyclic graphs (DAG) depicting the causal relationships between sleep apnoea and lung cancer**

**
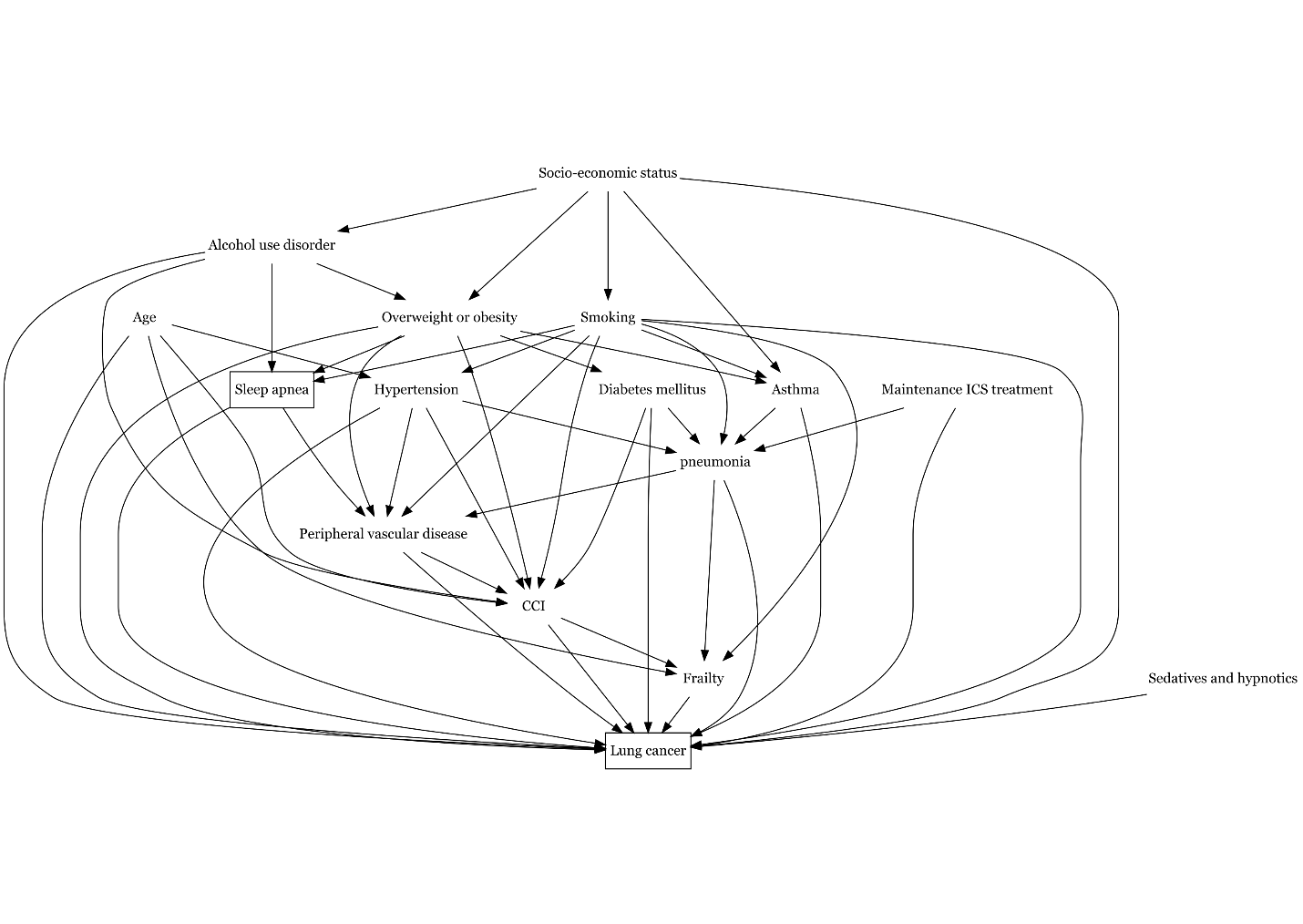
**

The direct acyclic diagram shows the causal relationship between sleep apnoea and lung cancer. CCI: Charlson comorbidity index; ICS: inhaled corticosteroids

**eFigure 3: The univariate hazard ratio of sleep apnoea on lung cancer, in COPD patients.**

**
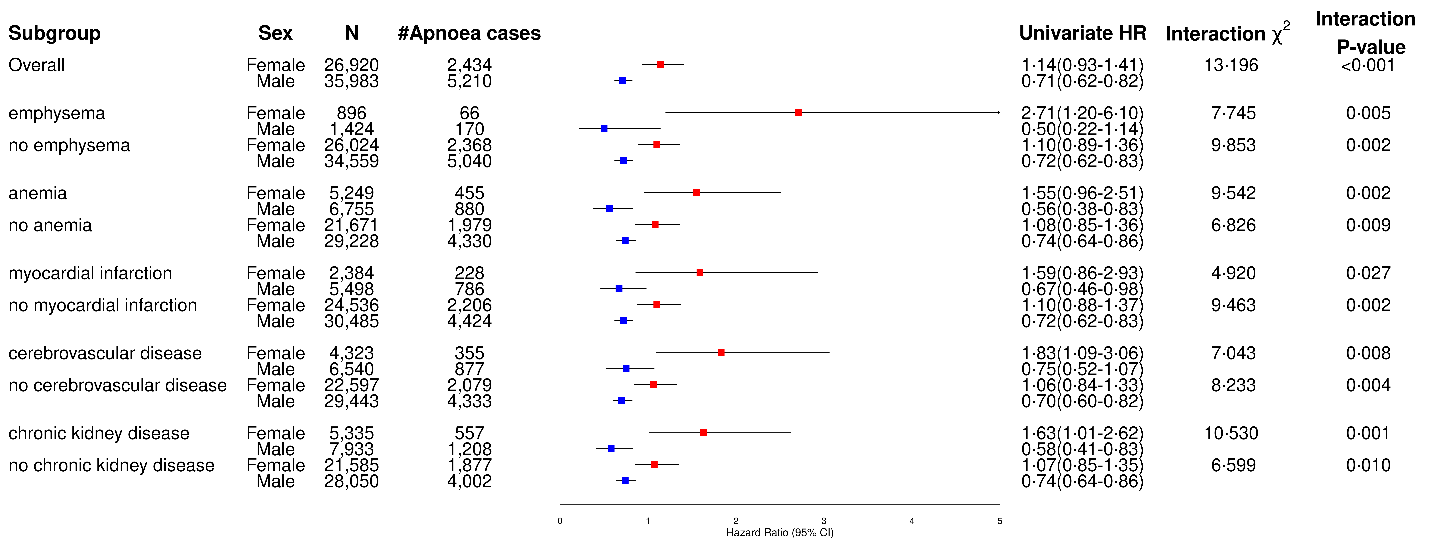
**

The sex-specific univariate hazard ratio of sleep apnoea on incident lung cancer, in COPD patients. A sex-specific stratification is shown with females (red), and males (blue) for the association in the overall study population and in specific subgroups based on the presence of hypoxia-related comorbidities. The 𝝌-squared and p-values present the significance of the interaction between sexes and sleep apnoea on lung cancer hazard within each group.

**eFigure 4:** **Sensitivity analyses of the multivariate hazard ratio of sleep apnoea on lung cancer, in COPD patients. Excluding incident sleep-apnoea**

**
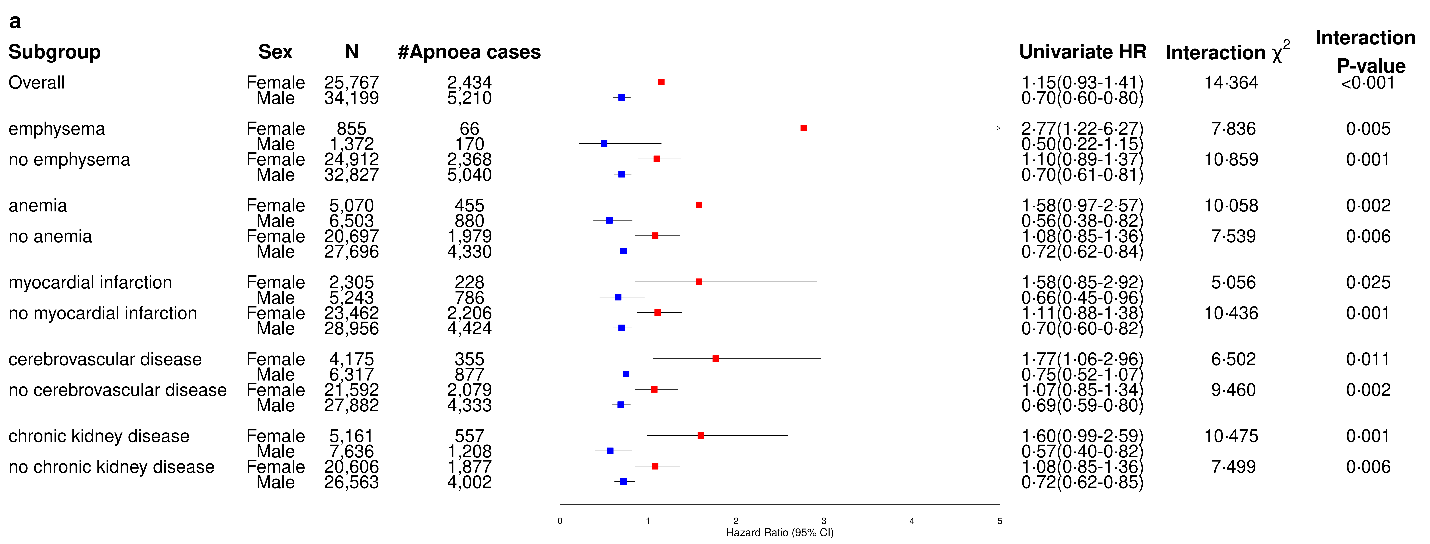
**

**
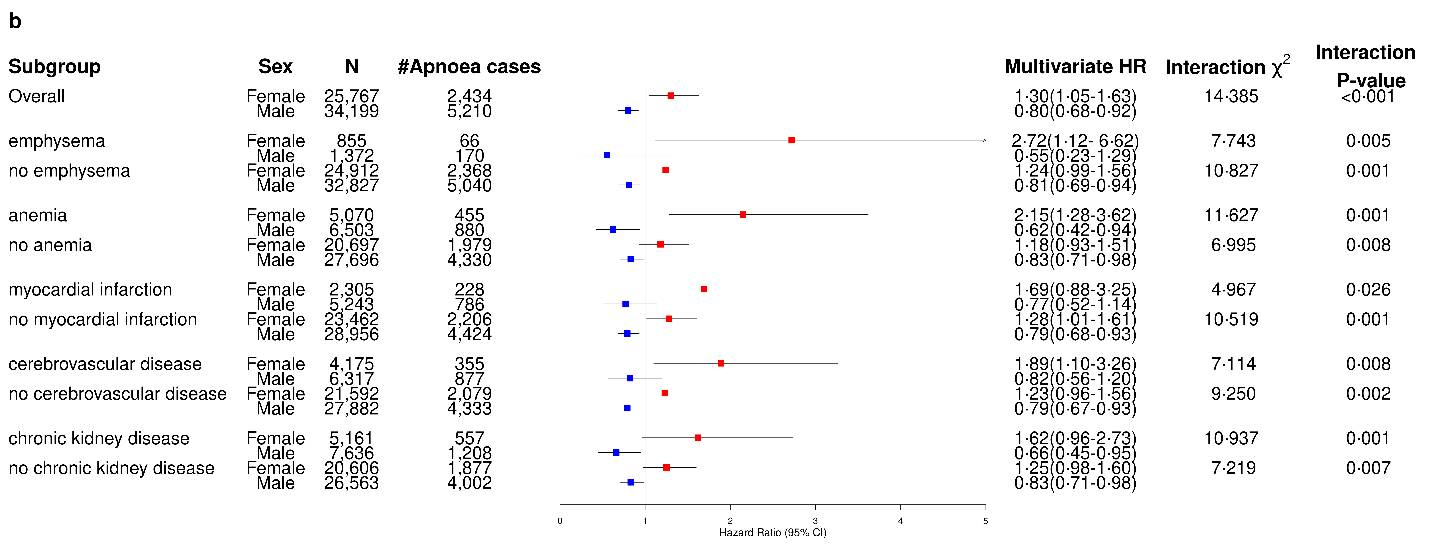
**

The sex-specific hazard ratio of sleep apnoea on incident lung cancer, in COPD patients. With the exclusion of patients with an incident sleep-apnoea diagnosis during follow-up. A sex-specific stratification is shown with females (red), and males (blue) for the association in the overall study population and in specific subgroups based on the presence of hypoxia-related comorbidities. The 𝝌-squared and p-values present the significance of the interaction between sexes and sleep apnoea on lung cancer hazard within each group. (A) shows the univariate hazard ratio. (B) shows the hazard ratio, adjusted for age, socioeconomic status, smoking status, alcoholism, overweight or obesity, hypertension, diabetes, asthma, pneumonia, peripheral vascular disease, Charlson comorbidity index, frailty, the use of inhaled corticosteroids, sedatives or hypnotic drugs.

**eFigure 5:** **Sensitivity analyses of the multivariate hazard ratio of sleep apnoea on lung cancer, in COPD patients.**

**
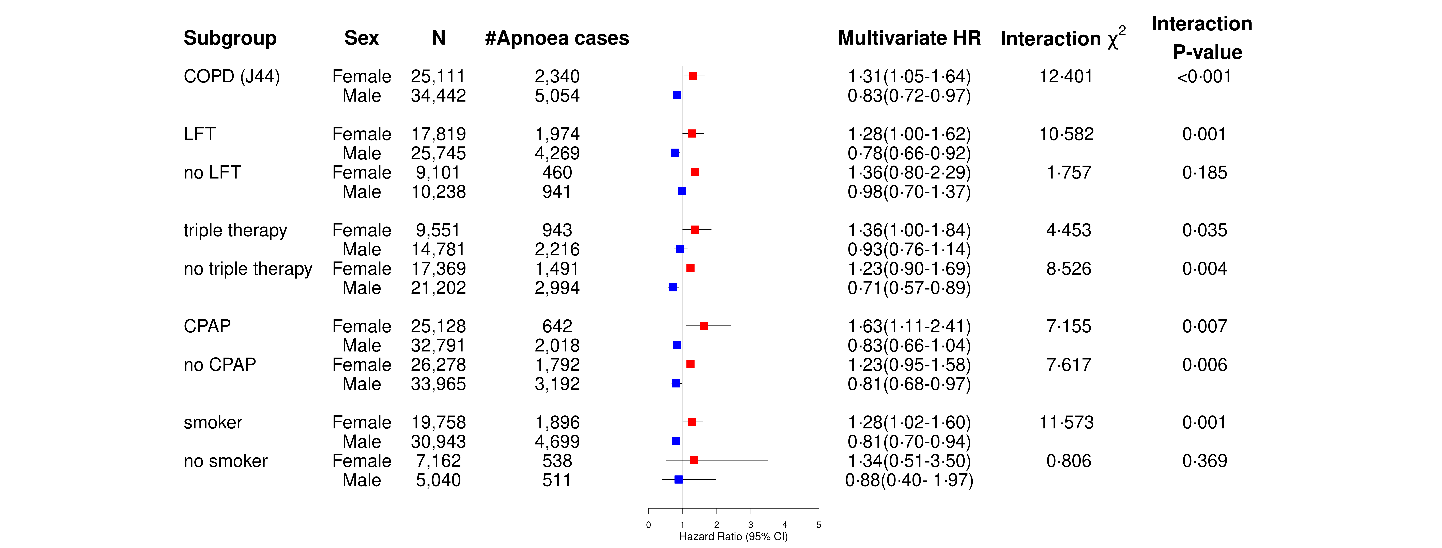
**

The sex-specific hazard ratio of sleep apnoea on incident lung cancer, in COPD patients. A sex-specific stratification is shown with females (red), and males (blue) for the association in specific subgroups: People who received a J44 ICD-10 diagnosis code, who performed and did not perform a lung function test, who received and did not recieve triple therapy, excluding people without a CPAP diagnosis and sleep apnoea, excluding people who received CPAP treatment, including only people who ever smoked and never smoked, including severe smokers (people who smoked and suffered from peripheral vascular disease) and excluding this group. The 𝝌-squared and p-values present the significance of the interaction between sexes and sleep apnoea on lung cancer hazard within each group. The hazard ratio is adjusted for age, socioeconomic status, smoking status, alcoholism, overweight or obesity, hypertension, diabetes, asthma, pneumonia, peripheral vascular disease, Charlson comorbidity index, frailty, the use of inhaled corticosteroids, sedatives or hypnotic drugs, excluding the factor where it is stratified on.

**References**

[1] Elm E von, Altman DG, Egger M, Pocock SJ, Gøtzsche PC, Vandenbroucke JP. The Strengthening the Reporting of Observational Studies in Epidemiology (STROBE) statement: guidelines for reporting observational studies. *The Lancet*. 2007;**370(9596)**:1453–1457. doi:10.1016/S0140-6736(07)61602-X

[2] Denholm R, Schüz J, Straif K, et al. Is Previous Respiratory Disease a Risk Factor for Lung Cancer? *Am J Respir Crit Care Med*. 2014;**190(5)**:549–559. doi:10.1164/rccm.201402–0338OC

[3] Schabath MB, Cote ML. Cancer Progress and Priorities: Lung Cancer. *Cancer Epidemiol Biomark Prev Publ Am Assoc Cancer Res Cosponsored Am Soc Prev Oncol*. 2019;**28(10)**:1563–1579. doi:10.1158/1055-9965.EPI-19-0221

[4] Cheong AJY, Tan BKJ, Teo YH, et al. Obstructive Sleep Apnoea and Lung Cancer: A Systematic Review and Meta-Analysis. *Ann Am Thorac Soc*. 2022;**19(3)**:469–475. doi:10.1513/AnnalsATS.202108-960OC

[5] Lee JY, Jeon I, Lee JM, Yoon JM, Park SM. Diabetes mellitus as an independent risk factor for lung cancer: A meta-analysis of observational studies. *Eur J Cancer*. 2013;**49(10)**:2411–2423. doi:10.1016/j.ejca.2013.02.025

[6] Lindgren A, Pukkala E, Nissinen A, Tuomilehto J. Blood Pressure, Smoking, and the Incidence of Lung Cancer in Hypertensive Men in North Karelia, Finland. *Am J Epidemiol*. 2003;**158(5)**:442–447. doi:10.1093/aje/kwg179

[7] Villemur B, Roux C, Poggi JN, Elias A, Le Hello C. Is it justified to search for cancer in patients with peripheral arterial disease? *JMV-J Médecine Vasc*. 2022;**47(3)**:133–140. doi:10.1016/j.jdmv.2022.07.003

[8] Lee, Y. M., Kim, S. J., Lee, J. H., & Ha, E. Inhaled corticosteroids in COPD and the risk of lung cancer. *International Journal of Cancer.* 2018*;***143(9):**2311–2318. doi:10.1002/ijc.31632
